# Comparison of clinical outcomes and perinatal outcomes between natural cycle and hormone replacement therapy of frozen-thawed embryo transfer in patients with regular menstruation: a propensity score-matched analysis

**DOI:** 10.3389/fendo.2024.1416841

**Published:** 2024-07-18

**Authors:** Lin Sun, Beining Yin, Zhiyi Yao, Congli Zhang, Jinyu Li, Sichen Li, Yueyue Cui, Fang Wang, Wei Dai, Zhiqin Bu, Yile Zhang

**Affiliations:** ^1^ Reproductive Medicine Center, The First Affiliated Hospital of Zhengzhou University, Zhengzhou, Henan, China; ^2^ Henan Key Laboratory of Reproduction and Genetics, First Affiliated Hospital of Zhengzhou University, Zhengzhou, Henan, China

**Keywords:** hormone replacement therapy, natural cycle, pregnancy outcomes, propensity score matching analysis, predictive model

## Abstract

**Purpose:**

To investigate potential differences in pregnancy outcomes among patients with regular menstruation who underwent frozen-thawed embryo transfer using natural cycle (NC) or hormone replacement therapy (HRT).

**Methods:**

This study retrospectively analyzed 2672 patients with regular menstruation who underwent FET from November 2015 to June 2021 at the single reproductive medical center. A one-to-one match was performed applying a 0.02 caliper with propensity score matching. Independent factors influencing the live birth and clinical pregnancy rates were screened and developed in the nomogram by logistic regression analysis. The efficacy of live birth rate and clinical pregnancy rate prediction models was assessed with the area under the ROC curve, and the live birth rate prediction model was internally validated within the bootstrap method.

**Results:**

The NC protocol outperformed the HRT protocol in terms of clinical pregnancy and live birth rates. The stratified analysis revealed consistently higher live birth and clinical pregnancy rates with the NC protocol across different variable strata compared to the HRT protocol. However, compared to the HRT treatment, perinatal outcomes indicated that the NC protocol was related to a higher probability of gestational diabetes. Multifactorial logistic regression analysis demonstrated independent risk factors for live birth rate and clinical pregnancy rate. To predict the two rates, nomogram prediction models were constructed based on these influencing factors. The receiver operating characteristic curve demonstrated moderate predictive ability with an area under curve (AUC) of 0.646 and 0.656 respectively. The internal validation of the model for live birth rate yielded an average AUC of 0.646 implying the stability of the nomogram model.

**Conclusion:**

This study highlighted that NC yielded higher live birth and clinical pregnancy rates in comparison to HRT in women with regular menstruation who achieved successful pregnancies through frozen-thawed embryo transfer. However, it might incur a higher risk of developing gestational diabetes.

## Introduction

The publication of the initial case of frozen-thawed embryo transfer (FET) in 1983 marked a significant milestone (1). Over the past four decades, the utilization of embryo freezing has steadily increased in China, Europe, and the United States, with frozen cycles accounting for over 40% of total cycles (2). Moreover, a comprehensive clinical multi-center study has discovered that FET substantially improves the live birth rate and reduces the probability of ovarian hyperstimulation syndrome (OHSS) versus fresh-cycle transfer (3). OHSS is an iatrogenic disease caused by overstimulation of the ovaries with exogenous gonadotropins during ovulation induction.

Endometrial preparation plays a crucial role in FET as it determines endometrial receptivity and coordinates the development of both the endometrium and embryo (4). Currently, numerous kinds of endometrial preparation protocols are employed for FET including the natural cycle, hormone replacement therapy, and promoting ovulation cycle with the NC and HRT being the most commonly used. Consequently, our objective was to discover the most beneficial endometrial preparation method for patients utilizing frozen embryos.

To date, multiple studies have endeavored to explore the clinical results of various endometrial preparation protocols, yet the conclusions remain inconsistent and controversial. Notably, when compared to HRT, NC has been revealed to offer a higher opportunity of live birth in young patients with regular cycles of menstruation (5, 6). However, several have reported that NC and HRT have similar outcomes and are equally effective (7, 8). Meanwhile, the repercussions of these two procedures on prenatal and neonatal outcomes have been the subject of a multitude of research. Retrospective investigations have indicated that there is a higher chance of adverse perinatal outcomes when receiving HRT (9–12). Conversely, Saito et al. demonstrated that the risk of gestational diabetes mellitus (GDM) was lower in HRT pregnancies (11). Consequently, there is no consensus regarding the safety and efficiency of the two endometrial preparation protocols.

In summary, the objective of this research project was to examine, the clinical and perinatal results between the NC and HRT protocols after FET in infertile patients with regular menstruation. The findings might provide valuable guidance to clinicians in selecting individualized protocols for FET patients.

## Materials and methods

### Study participants

This cohort study reviewed clinical information of patients receiving frozen-thawed cycles from November 2015 to June 2021 at the Reproductive Medicine Center of the First Affiliated Hospital of Zhengzhou University. The anonymous data were gathered from our center’s data entry systems. This research was approved by the hospital’s Institutional Review Board and Ethics Committee (reference number: 2023-KY-1115–002). On account of the study being retrospective, informed permission was not required. Every procedure was executed in compliance with applicable rules and legislation.

The research included individuals who fulfilled the subsequent criteria: 1) adoption of either the HRT or NC protocol; 2) compliance with the 2017 American Society for Reproductive Medicine consensus diagnostic criteria for infertility (13). The following were criteria for exclusion: 1) patients with other factors affecting pregnancy like ovarian insufficiency, adenomyosis, endometriosis, uterine abnormalities, uterine adhesions, cervical insufficiency or hydrosalpinx; 2) chromosomal abnormalities in the patient or spouse; 3) recurrent implantation failures; 4) abnormal male reproductive function; 5) irregular menstrual cycles (<21 or >35 days); 6) incomplete clinical information.

### Study procedures

#### Endometrial preparation protocols

Every method performed to prepare the endometrium for this research was meticulously documented. Patients were allocated to either the NC group or the HRT group based on their individual circumstances and the experience of the clinician.

### NC protocol

For patients in the NC group, on the eighth and ninth day of the menstrual cycle, transvaginal ultrasounds were implemented to monitor follicular development and endometrial growth. Once the dominant follicle reached 14 mm in mean diameter, transvaginal ultrasounds and the level of urinary luteinizing hormone (LH) level were monitored every day. On the day of ovulation (Day 1, D1), 400mg of vaginal progesterone soft capsules (Utrogestan, Cyndea Pharma, S.L, Spain) was administered once daily, and three days later, oral dydrogesterone (Duphaston; Abbott, Netherlands) was implemented. In accordance with the fact that the optimal endometrial receptivity usually occurs between the fourth and sixth days after ovulation, cleavage-stage embryos and blastocysts were implanted on D4 and D5 respectively (14).

### HRT protocol

For patients undergoing the HRT protocol, estradiol valerate 4mg (Progynova^®^; Bayer, Leverkusen, Germany) was taken every day beginning on the menstrual cycle’s third day. Every four days, the oral dosage was modified based on the thickness of the endometrium. Once the endometrium thickness reached 7mm, intramuscular progesterone (60mg) was administered to the protocol to transform the endometrium (Day 1, D1). The following day, a dose of 10 mg/day of oral dydrogesterone was applied and the dose was raised to 30 mg/day after three days. Cleavage-stage embryos and blastocysts were transferred on D5 and D6 respectively.

### Embryo selection and evaluation

We selected one or two good-quality blastocyst and cleavage embryos to transfer for each patient. The following are the criteria for transfer. Embryos were evaluated according to Peter’s criteria: grade I and grade II were considered high quality (15). Blastocysts were scored according to the Gardner criteria: high-quality embryos are those with 2 scores of B and above for inner cell mass and trophectoderm (16).

### Luteal phase support and confirmation of pregnancy

Under the supervision of an ultrasonography, up to two embryos were implanted into the uterus. Starting from the day of embryo transplantation, daily administration of 90mg progesterone sustained-release vaginal gel (Crinone 8%; Merck Serono, Switzerland) or 400mg progesterone soft capsules and 20mg of oral dydrogesterone was initiated. But in HRT protocol, 10mg estradiol valerate is required in addition to the above medications. The serum human chorionic gonadotropin (hCG) levels were tested two weeks following the transfer of the embryo. Upon surpassing 50 IU/L in blood hCG, the luteal phase was continued. In the fifth week following the embryo transfer, transvaginal ultrasound was conducted to clinically confirm pregnancy. If pregnancy occurred, the luteal phase was still continued. Progesterone sustained-release vaginal gel or progesterone soft capsules was discontinued in the 45th day after transportation and oral dydrogesterone was discontinued in the 65th day after transportation.

### Definition of clinical outcomes

Every patient received follow-up for no fewer than a year. Live birth was defined as the delivery of at least one live child beyond 22 weeks of pregnancy. Clinical pregnancy was defined as the presence of one or more gestational sacs observed by ultrasonography or the presence of clear clinical indicators of pregnancy. A preterm birth occurred after 22 weeks but before the full 37 weeks of gestation. Low-birth-weight infants and macrosomia were defined as those with a birth weight of less than 2,500 g and a weight of more than 4,000 g respectively.

### Statistical analysis

This study was statistically analyzed using SPSS 25.0 software and the R language software statistical package (R version 4.1.3). Dichotomous variables were expressed as percentages (%), while mean ± standard deviation is the presentation format for continuous variables with a normally distributed distribution. Comparisons of two independent samples of dichotomous variables were evaluated using the chi-square test, and Fisher’s exact test or chi-square test was employed to assess the count data. For normally distributed continuous variables that met the assumption of equal variances, the independent t-test was employed. Propensity score matching (PSM) was employed to 1:1 match baseline data with statistically significant differences within either of the groups with a caliper value of 0.02. The variables used for matching included female age, infertility duration, infertility type, gravity, parity, NO. of miscarriages, BMI, basal serum FSH, AMH, AFC, type of embryo transferred and NO. of embryo transferred. The study performed univariate logistic regression analysis to ascertain independent and confounding factors. Moreover, the multivariate logistic regression analysis incorporated variables from the univariate study that were correlated to the two clinical outcomes. The study conducted stratified analyses based on female age, infertility duration, BMI, AFC, the type of embryo transferred, number of embryos transferred, endometrial thickness, and triple-line endometrial pattern on the day of progesterone administration, to observe the effects of the two protocols in different subgroup.

The independent variables affecting the rates of clinical pregnancy and live births were identified by the multifactorial logistic regression analysis. Incorporating these factors as modeling variables, predictive nomogram models were built using the R statistical software. The bootstrap sampling method was employed for internal validation of the model, and the predictive power of the model was assessed by calculating the area under the curve (AUC) and the receiver operating characteristic (ROC) curve. The statistical significance was identified by applying a two-sided significance criterion of 0.05. **Supplementary Figure 1** illustrated the study’s data collection methodology.

## Results

The research comprised 3,569 patients who underwent FET between November 2015 and June 2021. Of those, 1,914 patients were in the HRT group and 1,655 individuals belonged to the NC group. After conducting propensity score-matched (PSM), 1,336 infertile patients with regular menstruation were included in each group.

### Baseline characteristics and clinical outcome characteristics before and after matching

Baseline characteristics were illustrated in **Table 1**. Before matching, the NC and HRT groups had statistically significant differences in terms of female age, infertility duration, infertility type, gravity, parity, number of miscarriages, BMI, AMH, number of AFC, number of embryos transferred, and type of embryos transferred. Following PSM, all these variables were balanced between the two groups. The clinical outcomes before and after matching were also presented in **Table 1**. Before PSM, the NC group exhibited better endometrial thickness and higher incidence of live birth and clinical pregnancy and single pregnancy. After PSM, these differences remained significant, with the NC group consistently exhibiting higher rates of live birth, clinical pregnancy and thicker endometrium versus the HRT group.

**Table 1:** Comparison of baseline characteristics and pregnancy outcomes between NC and HRT before and after PSM.

Characteristics	Before propensity score matching (n = 3,569)			After propensity score matching (n = 2,672)		
	NC (n = 1,655)	HRT (n = 1,914)	P value	NC (n = 1,336)	HRT (n = 1,336)	P value
Female age (y)	31.25 ± 4.60	30.38 ± 4.80	<0.001	31.10 ± 4.60	30.95 ± 4.89	0.408
Infertility duration (y)	4.20 ± 3.16	3.90 ± 2.94	0.003	4.13 ± 3.04	4.04 ± 3.13	0.471
Infertility type, n (%)			<0.001			0.485
Primary	733 (44.29)	1,013 (52.93)		612 (45.81)	630 (47.16)	
Secondary	922 (55.71)	901 (47.07)		724 (54.19)	706 (52.84)	
Gravidity	0.96 ± 1.18	0.81 ± 1.15	<0.001	0.93 ± 1.16	0.92 ± 1.21	0.782
Parity	0.27 ± 0.50	0.19 ± 0.44	<0.001	0.24 ± 0.47	0.22 ± 0.47	0.306
No. of miscarriages	0.46 ± 0.76	0.40 ± 0.79	0.026	0.46 ± 0.76	0.46 ± 0.82	0.903
BMI (kg/m^2^)	22.32 ± 2.89	22.76 ± 3.20	<0.001	22.47 ± 2.93	22.59 ± 3.17	0.284
Basal serum FSH (mIU/ml)	6.71 ± 1.84	6.84 ± 21.27	0.812	6.61 ± 1.81	6.51 ± 1.89	0.154
AMH (ng/ml)	3.49 ± 2.36	4.35 ± 3.03	<0.001	3.63 ± 2.42	3.71 ± 2.33	0.376
AFC	14.40 ± 5.73	16.18 ± 6.04	<0.001	15.01 ± 5.71	15.37 ± 5.98	0.113
No. of embryos transferred, n(%)			<0.001			0.431
1	692 (41.81)	692 (36.15)		535 (40.04)	555 (41.54)	
2	963 (58.19)	1,222 (63.85)		801 (59.96)	781 (58.46)	
Type of embryo transferred, n(%)			0.187			0.333
Cleavage stage	854 (51.60)	1,030 (53.81)		695 (52.02)	670 (50.15)	
Blastocyst stage	801 (48.40)	884 (46.19)		641 (47.98)	666 (49.85)	
Endometrial thickness on the day of progesterone administration (mm)	10.33 ± 1.93	9.62 ± 1.64	<0.001	10.24 ± 1.92	9.56 ± 1.61	<0.001
Triple-line endometrial pattern on the day of progesterone administration, n(%)			<0.001			<0.001
B	1,330 (80.36)	1,814 (94.78)		1,080 (80.84)	1,267 (94.84)	
B-C	146 (8.82)	34 (1.78)		126 (9.43)	29 (2.17)	
C	179 (10.82)	66 (3.45)		130 (9.73)	40 (2.99)	
Biochemical pregnancy rate, n(%)	1,020 (61.63)	1,130 (59.04)	0.114	828 (61.98)	761 (56.96)	0.008
Clinical pregnancy rate, n(%)	957 (57.82)	1,031 (53.87)	0.018	779 (58.31)	683 (51.12)	<0.001
Live-birth rate, n(%)	797 (48.16)	849 (44.36)	0.023	642 (48.05)	551 (41.24)	<0.001
Single pregnancy rate, n(%)	625 (37.76)	659 (34.43)	0.039	500 (37.43)	437 (32.71)	0.011
Twin pregnancy rate, n(%)	172 (10.39)	190 (9.93)	0.646	142 (10.63)	114 (8.53)	0.066
Miscarriage rate, n(%)	140 (8.46)	162 (8.46)	0.996	118 (8.83)	119 (8.91)	0.946
Ectopic pregnancy rate, n(%)	16 (0.97)	21 (1.10)	0.701	16 (1.20)	14 (1.05)	0.713

AFC, antral follicle count; AMH, antimullerian homone; BMI, body mass index; FSH, follicle-stimulating hormone; HRT, hormone replacement therapy; NC, natural cycle; PSM, propensity score matching.

### Relationship and stratification of endometrial preparation protocols with live birth rate and clinical pregnancy rate

The influence of different variables was evaluated utilizing univariate and multivariate analysis (**Supplementary Table 1**). The results revealed that female age, infertility duration, and the endometrial preparation protocol were associated with the live birth rate and clinical pregnancy rate negatively. Conversely, AFC, NO. of embryos transferred, type of embryos transferred, and endometrial thickness on the day of progesterone administration were positively associated with them. To ascertain whether there was a consistent correlation across several subgroups between the live birth rate and various endometrial preparation protocols, stratified analyses were performed based on female age, infertility duration, BMI, AFC, the type of embryo transferred, number of embryo transferred, endometrial thickness and triple-line endometrial pattern on the day of progesterone administration (**Table 2**). The NC group had an increased chance of achieving clinical pregnancy and live births in the subgroup that female age ≤35 years, BMI ≤24kg/m^2^, AFC >15, the number of embryos transferred was one, transferred any embryo type, the endometrial thickness <10mm, and B endometrial pattern. It appeared that there was no interaction between any of the subgroups (p > 0.05).

**Table 2:** Impact of two endometrial preparation protocols on live birth rate and clinical pregnancy rate in each subgroup^a^.

Subgroups	No. of patients	Live birth rate			Clinical pregnancy rate		
		OR(95% CI)	P value	P for interaction	OR(95% CI)	P value	P for interaction
Female age (y)				0.225			0.298
≤35	2,211	0.80 (0.67,0.96)	0.019		0.79 (0.66,0.95)	0.010	
>35	461	0.63 (0.40,0.97)	0.038		0.73 (0.44,1.22)	0.227	
Infertility duration (y)				0.186			0.351
≤3	1,460	0.68 (0.54,0.86)	0.001		0.72 (0.57,0.90)	0.004	
>3	1,212	0.87 (0.68,1.12)	0.283		0.86 (0.67,1.11)	0.247	
BMI(kg/m2)				0.502			0.420
≤24	1,949	0.75 (0.62,0.91)	0.004		0.75 (0.62,0.91)	0.003	
>24	723	0.81 (0.58,1.12)	0.200		0.86 (0.62,1.20)	0.386	
AFC				0.498			0.629
≤15	1,376	0.83 (0.66,1.05)	0.121		0.81 (0.64,1.03)	0.090	
>15	1,296	0.70 (0.55,0.89)	0.004		0.74 (0.58,0.93)	0.011	
No. of embryos transferred				0.095			0.279
1	1,090	0.65 (0.49,0.84)	0.001		0.70 (0.53,0.91)	0.008	
2	1,582	0.86 (0.69,1.06)	0.157		0.84 (0.67,1.03)	0.098	
Type of embryo transferred				0.187			0.936
Cleavage stage	1,365	0.85 (0.67,1.07)	0.168		0.79 (0.62,1.00)	0.049	
Blastocyst stage	1,307	0.68 (0.54,0.87)	0.002		0.76 (0.60,0.96)	0.022	
Endometrial thickness on the day of progesterone administration				0.590			0.800
≤10	1,798	0.73 (0.59,0.89)	0.002			0.018	
>10	874	0.81 (0.61,1.09)	0.165		0.75 (0.56,1.00)	0.051	
Triple-line endometrial pattern on the day of progesterone administration				0.737			0.378
B	2,347	0.75 (0.63,0.90)	0.001		0.80 (0.67,0.95)	0.011	
B-C	155	1.07 (0.44,2.59)	0.881		0.69 (0.29,1.62)	0.397	
C	170	0.84 (0.38,1.86)	0.661		0.52 (0.23,1.17)	0.112	

AFC, antral follicle count; AMH, antimullerian homone; BMI, body mass index; CI, confidence interval; FSH, follicle-stimulating hormone; OR, odds ratio.

^a^Adjusted for female age, infertility duration, infertility type, AFC, BMI, FSH, AMH, endometrial thickness, number of transferred embryos, type of embryo transferred, triple-line endometrial pattern.

### Adverse pregnancy outcomes and perinatal outcomes

There was a substantial increase in the likelihood of gestational diabetes when comparing the two protocols (**Table 3**). However, there were no statistical differences observed in gestational hypertension, premature rupture of membranes, or other adverse outcomes, including anemia during pregnancy, oligohydramnios, meconium stained amniotic fluid, fetal distress, and placental abruption. Among the 1,193 newborns involved in the research, no statistically significant differences existed within the two groups in preterm birth rate, newborn weights, cesarean section rate, or weights of single and twin pregnancies.

**Table 3:** Perinatal outcomes and neonatal outcomes based on endometrial preparation protocols.

Characteristics	NC(n = 1336)	HRT(n = 1336)	P value
PTB, n(%)	85 (6.36)	79 (5.91)	0.629
Cesarean section rate, n(%)	466 (34.88)	423 (31.66)	0.077
Perinatal outcomes			
GDM, n(%)	23 (1.72)	11 (0.82)	0.038
HDP, n(%)	27 (2.02)	33 (2.47)	0.433
pPROM, n(%)	24 (1.80)	18 (1.35)	0.351
Others, n(%)	26 (1.95)	25 (1.87)	0.888
Single pregnancy			
Birthweight (g)	3,395.02 ± 468.59	3,431.62 ± 528.82	0.262
LBW, n(%)	19 (1.42)	15 (1.12)	0.490
Macrosomia, n(%)	30 (2.25)	42 (3.14)	0.152
Twin pregnancy			
Higher weight(g)	2,803.06 ± 401.45	2,762.89 ± 496.35	0.475
Lower weight(g)	2,494.65 ± 406.86	2,434.53 ± 422.04	0.249
All weight ≤2500, n(%)	21 (1.57)	18 (1.35)	0.628
All weight >2500, n(%)	83 (6.21)	64 (4.79)	0.107

HDP, hypertensive disorders of pregnancy; HRT, hormone replacement therapy; GDM, gestational diabetes mellitus; LBW, low birthweight; NC, natural cycle; pPROM, preterm premature rupture of the membrane; PTB, preterm birth.

Others: anemia during pregnancy, oligohydramnios, meconium stained amniotic fluid, fetal distress, and placental abruption.

### Construction and internal validation of the predictive model for nomogram

**Figure 1:**
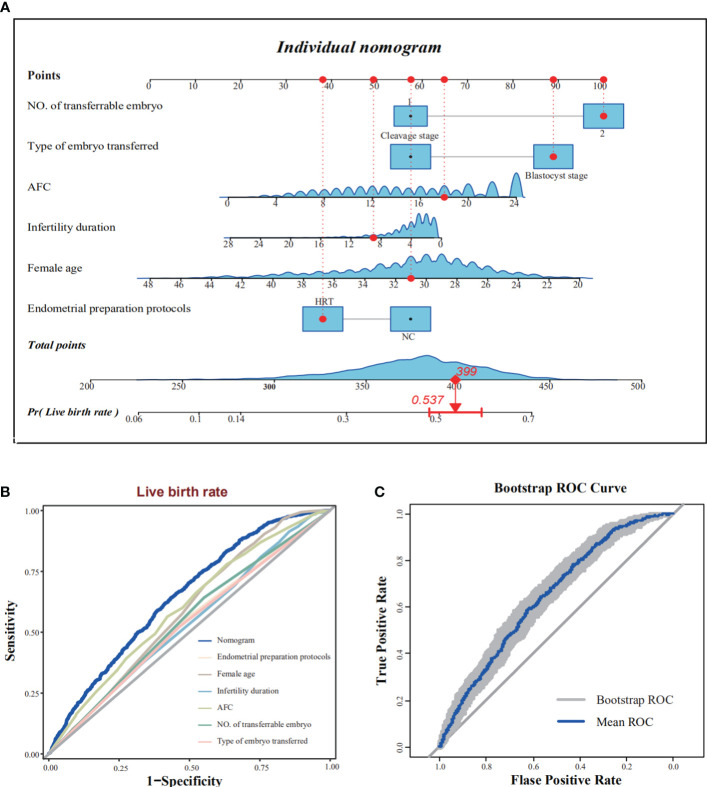
Construction and internal validation of the predictive model for live birth rate. **(A)** The nomogram exhibited six characteristics of a patient (NO. of transferrable embryo = 2, Type of embryo transferred = Blastocyst stage, AFC = 18, Infertility duration = 9, Female age = 31, Endometrial Preparation Protocol = HRT), with a total score of 399 points, and the predicted probability of live birth was 53.7%. **(B)** The area under the curve of the nomogram model was 0.646 (95% CI: 0.626–0.667), indicating medium predictive power. **(C)** Internal validation with 1000 repeated sampling was carried out to predict the effectiveness of the model, resulting in an average AUC = 0.646 (95% CI: 0.629–0.663). AFC, antral follicle count; AUC, area under the curve; CI, confidence interval; HRT, hormone replacement therapy; NC, natural cycle; ROC, receiver operating characteristic curve.

The independent variables influencing the live birth rate were uncovered by the multifactorial logistic regression analysis. The results revealed that the endometrial preparation protocol, female age, infertility duration, AFC, type of embryos transferred and number of embryos transferred were significant factors (**Figure 1A**). The predictive model for the live birth rate was evaluated using the ROC curve, and an area under the curve of 0.646 (95% CI: 0.626–0.667) was obtained, indicating a moderate predictive power. Moreover, the predictive ability of the nomogram model with that of each individual indicator was also compared using ROC curves (**Figure 1B**). The nomogram model outperformed each indicator in terms of predictive ability. In addition, A computer simulation of repeated sampling was executed to further validate the model internally. The ROC curve was employed for calculating the model’s predictive performance after 1000 repeated samples, resulting in an average AUC = 0.646 (95% CI: 0.629–0.663) (**Figure 1C**). The AUC remained essentially unchanged after internal validation, indicating the stability of the model. Additionally, we also constructed the nomogram prediction model for the clinical pregnancy rate and leveraged the ROC curve with the AUC of 0.656 (95% CI: 0.635–0.677). (**Supplementary Figure 2**)

## Discussion

The FET cycle has grown in favor all around the world in light of its ability to reduce the incidence of ovarian stimulation syndrome, preserve female fertility, and provide other advantages (17, 18). Consequently, research on the effects of various endometrial preparation protocols on the result of pregnancy is becoming more and more popular. Our investigation demonstrated that the NC protocol produced greater rates of clinical pregnancy and live births.

Currently, the most appropriate protocol for endometrial preparation is still no consensus. Our observations are substantiated by recent published articles. Within the patient subgroup with D5/D6 blastocyst embryo transfer, the NC group revealed a trend toward greater clinical pregnancy and live birth rates, according to a large retrospective cohort analysis (19). Two other studies reached similar conclusions (20, 21). Another study with low-quality evidence elucidated that for double embryo transfer, the modified NC group had dramatically superior clinical outcomes compared to the HRT group (22). However, several relevant studies displayed no distinction in clinical outcomes within the two protocols (23–26). Consistent conclusions have also been presented in two high-quality Cochrane analyses. Ghobara et al. presumed that in patients with regular menstruation but low fertility, there was inadequate data to prove the superiority of one endometrial preparation protocol over another (27). Similarly, Glujovsky et al. indicated a lack of evidence regarding specific interventions for endometrial preparation in patients receiving frozen embryo transfers (28). Nevertheless, in our research, all FET cycles were included for embryo transfer, and women with low fertility were excluded to minimize the interference of confounding factors. This might explain why the NC group in our cohort achieved optimal clinical outcomes.

In light of the underlying mechanism, it might be related to the type of embryo transferred. It has been warranted that fresh blastocyst transfers had a higher probability of live birth and clinical pregnancy than fresh cleavage stage transfers (29). While many studies prefer blastocyst transfers, the optimal endometrial preparation protocol for blastocyst transfers remains inconsistent (7, 20). Researchers are convinced that only surviving embryos will undergo self-selection, and blastocysts undergo a reselection program during the developmental block at the eight-cell stage to eliminate embryos with inadequate potential for development (30). Therefore, blastocyst transfer might be a superior choice for research purposes. However, there have also been cohort studies that revealed no disparity in cumulative live birth rate regarding the transfer of blastocysts and cleavage (31). In our study, no distinction was made between the cleavage stage and blastocyst embryos, but stratified analysis revealed that the NC protocol was inclined to achieve remarkable clinical benefits in both groups.

In addition, the embryo must be implanted in the endometrium at the “window of implantation”, which represents the period of highest receptivity for trophoblast-endometrium interactions (4). A study similar to ours transferred cleavage embryos one day earlier than our center but reached the same conclusion that the live birth rate increased as a result of the NC protocol (21). Another low-quality analysis performed with both cleavage and blastocyst transfers one day earlier than our center found no distinction between the two protocols about the clinical pregnancy outcomes (25). Other research has indicated that prolonged progesterone supplementation is linked to an increased incidence of biochemical pregnancy (32). However, the ideal length of time to use progesterone supplements remains a subject of debate, as prolonged supplementation might narrow the window of implantation, while a too-short period could raise the chance of losing a pregnancy too soon (32–35). Taken together, the intricate process of implantation comprises both intercellular and extracellular matrix interactions as well as spatiotemporally regulated endocrine, paracrine, and autocrine interactions (36). Furthermore, every center has a varied choice of when to schedule the transfer day depending on the width of the implantation window. At our center, despite developing two days apart, the blastocysts and cleavage stage embryos were transferred one day apart. This is due to the fact that our center accidentally discovered that transplanting one day apart may resulted in better pregnancy outcomes in 2016 (37–39).

Furthermore, endometrial preparation protocols might also impact obstetric and perinatal outcomes. Administering the HRT protocol might raise the risk of hypertensive disorders in pregnant women with preeclampsia and other hypertensive diseases (10, 40–42). According to a high-quality review, the NC protocol was related to lower rates of macrosomia, hypertensive disorders of pregnancy, and early pregnancy loss (43). Nevertheless, no differences in neonatal and perinatal outcomes were found within either of the groups in our research, which could be brought about by the tiny sample size. Additionally, we observed a higher risk of developing gestational diabetes mellitus (GDM) in the NC group, consistent with a study by Saito et al. (11). A review of the data has demonstrated that maternal peripheral insulin resistance is a critical event in the occurrence of GDM. The HRT protocol might reduce the release of insulin-resistant hormones from the placenta, potentially reducing the incidence of GDM (44). However, other studies have displayed no difference in the risk of developing GDM between the two groups (45, 46). Therefore, the placenta may also play an indispensable role in adverse neonatal and perinatal outcomes, necessitating further exploration in future large-scale studies.

Our study possesses several noteworthy strengths. Firstly, this study is the first PSM cohort study to analyze the effects of natural versus hormone replacement cycles on pregnancy outcomes and perinatal outcomes with a huge sample size used to guarantee robust statistical power. Additionally, the study population included people whose average was thirty years and was not limited by age, thereby enhancing the generalizability of the findings to a wide range of patients. Moreover, patients with regular menstruation were specifically selected to minimize selection bias. In addition, to control confounding factors, we implemented stratified analysis and logistic regression making conclusions more dependable. Finally, we internally validated the nomogram prediction model, and the ROC curves and AUC supported the model’s sensitivity and accuracy.

However, this research does have its limitations. To begin with, given that the study was a retrospective cohort, confounders besides the variables collected in the study could not be investigated, such as lifestyle habits (smoking or non-smoking) and adjustments made by clinicians during the study period. Secondly, the data were solely derived from the same center, and future studies should involve multiple centers for broader analysis and further prospective randomized controlled trials. Thirdly, the protocol criteria exclude most gynecological conditions that could potentially influence endometrial receptivity. Therefore, these results might not encompass patients with impaired endometrial receptivity. Fourthly, this analysis didn’t perform preimplantation genetic testing for aneuploidies (PGT-A) because of the restricted data accessibility, thus transfer failure due to aneuploid embryos could not be excluded. If euploid embryos were being transferred, the findings would be more robust.

## Conclusions

This study demonstrated that natural cycles yield higher rates of live birth and clinical pregnancy by contrasting hormone replacement cycles in women with regular menstruation undergoing frozen-thawed embryo transfer. However, it was vital to note that natural cycles were linked to a greater incidence of gestational diabetes. Based on our findings, we recommend the use of natural cycles as the preferred option for performing FET. However, it will take more superior prospective randomized controlled studies to identify and validate the most appropriate endometrial preparation strategy.

## Data availability statement

The raw data supporting the conclusions of this article will be made available by the authors, without undue reservation.

## Ethics statement

The studies involving humans were approved by Ethics Committee of Scientific Research and Clinical Trials of the First Affiliated Hospital of Zhengzhou University. The studies were conducted in accordance with the local legislation and institutional requirements. Written informed consent for participation was not required from the participants or the participants’ legal guardians/next of kin in accordance with the national legislation and institutional requirements.

## Author contributions

LS: Data curation, Formal analysis, Software, Writing – original draft. BY: Data curation, Software, Visualization, Writing – original draft. ZY: Writing – review & editing. CZ: Writing – review & editing. JL: Writing – review & editing. SL: Writing – review & editing. YC: Writing – review & editing. FW: Writing – review & editing. WD: Writing – review & editing. ZB: Writing – review & editing. YZ: Conceptualization, Data curation, Funding acquisition, Resources, Supervision, Writing – review & editing.

## Supplementary material

Supplementary Figure 1 The flowchart of participants. HRT, hormone replacement therapy; NC, natural cycle.

Supplementary Figure 2 Construction of the predictive model for clinical pregnancy rate. The nomogram revealed five characteristics of a patient (NO. of transferrable embryo = 2, Type of embryo transferred = Blastocyst stage, AFC = 18, Infertility duration = 9, Female age = 31, Endometrial Preparation Protocol = HRT), with a total score of 329 points, and the predicted probability of clinical pregnancy rate was 70.5%. The area under the curve of ROC for the nomogram model was 0.656 (95% CI: 0.635–0.677). AFC, antral follicle count; CI, confidence interval; HRT, hormone replacement therapy; NC, natural cycle; ROC, receiver operating characteristic curve.
