# Supplementary figures and images for: Comparison of clinical outcomes and perinatal outcomes between natural cycle and hormone replacement therapy of frozen-thawed embryo transfer in patients with regular menstruation: a propensity score-matched analysis
Source: Front Endocrinol (Lausanne). 2024 Jul 18;15:1416841. doi: 10.3389/fendo.2024.1416841 (PMC11291223; doi:10.3389/fendo.2024.1416841)

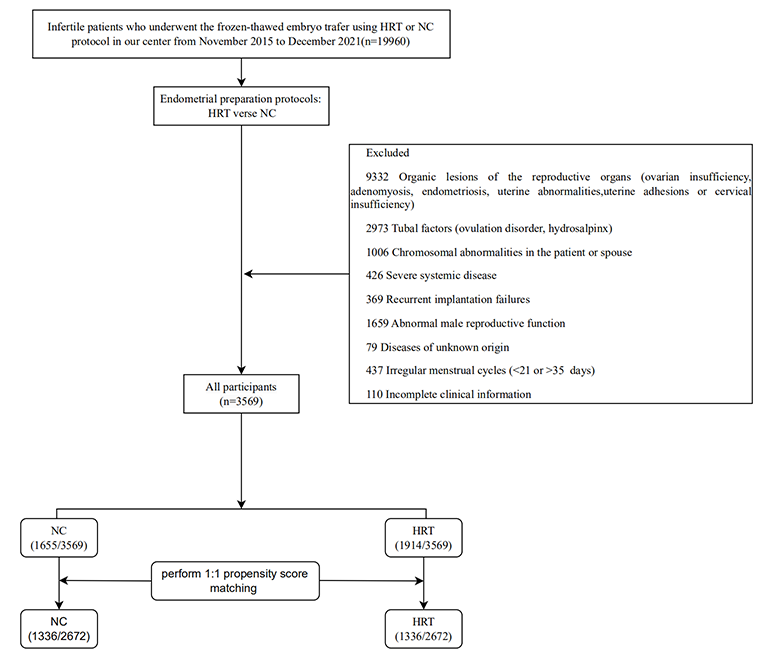

Supplement: Supplementary Figure 1 — The flowchart of participants. HRT, hormone replacement therapy; NC, natural cycle. [file Image_1.tif]

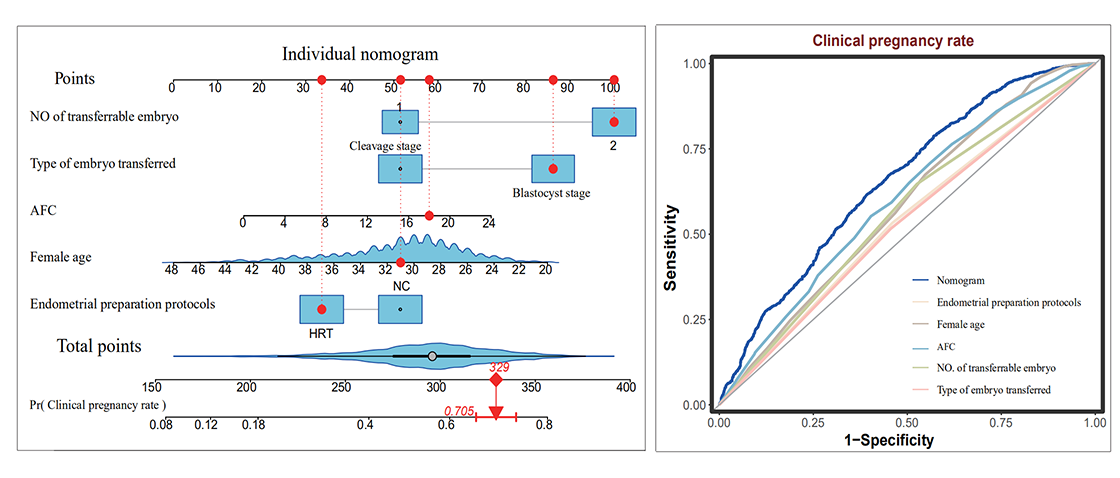

Supplement: Supplementary Figure 2 — Construction of the predictive model for clinical pregnancy rate. The nomogram revealed five characteristics of a patient (NO. of transferrable embryo = 2, Type of embryo transferred = Blastocyst stage, AFC = 18, Infertility duration = 9, Female age = 31, Endometrial Preparation Protocol = HRT), with a total score of 329 points, and the predicted probability of clinical pregnancy rate was 70.5%. The area under the curve of ROC for the nomogram model was 0.656 (95% CI: 0.635–0.677). AFC, antral follicle count; CI, confidence interval; HRT, hormone replacement therapy; NC, natural cycle; ROC, receiver operating characteristic curve. [file Image_2.tif]
